# Inhibitory Effects of STAT3 Transcription Factor by Synthetic Decoy ODNs on Autophagy in Renal Fibrosis

**DOI:** 10.3390/biomedicines9040331

**Published:** 2021-03-25

**Authors:** Young-Ah Kim, Hyun-Ju Kim, Mi-Gyeong Gwon, Hyemin Gu, Hyun-Jin An, Seongjae Bae, Jaechan Leem, Hyun Jin Jung, Kwan-Kyu Park

**Affiliations:** 1 Department of Pathology, School of Medicine, Catholic University of Daegu, Daegu 42472, Korea; youngah7840@naver.com (Y.-A.K.); bale4450@naver.com (H.-J.K.); daldy88@cu.ac.kr (M.-G.G.); guhm1207@cu.ac.kr (H.G.); ahj119@cu.ac.kr (H.-J.A.); zz22@cu.ac.kr (S.B.); 2 Department of Immunology, School of Medicine, Catholic University of Daegu, Daegu 42472, Korea; jcim@cu.ac.kr; 3 Department of Urology, School of Medicine, Catholic University of Daegu, Daegu 42472, Korea; hnjini@cu.ac.kr

**Keywords:** STAT3, decoy ODNs, autophagy, renal fibrosis, UUO

## Abstract

Autophagy in the proximal tubules may promote fibrosis by activating tubular cell death, interstitial inflammation, and the production of pro-fibrotic factors. The signal transducer and activator of transcription 3 (STAT3) is activated as a potential transcription factor, which mediates the stimulation of renal fibrosis. We investigated the role of the STAT3 in autophagy and its effect on the prevention of interstitial renal fibrosis. In this study, we use synthesized STAT3 decoy oligonucleotides (ODN), which were injected into the tail veins of unilateral ureteral obstruction (UUO) mice, to explore the regulation of autophagy in UUO-induced renal fibrosis. The expression of interleukin-6 (IL-6), interleukin-1β (IL-1β), tumor necrosis factor-α (TNF-α), and collagen were decreased by STAT3 decoy ODN. The autophagy markers microtubule-associated protein light chain 3 (LC3) and fibronectin, were identified through immunofluorescent staining, indicating that they were reduced in the group injected with ODN. The expressions of LC3, Beclin1, p62, and autophagy-related 5–12 (Atg5–12) and hypoxia inducible factor-1α (HIF-1α) were inhibited in the ODN injection group. We determined the inhibitory effect of autophagy in chronic kidney disease and confirmed that STAT3 decoy ODN effectively inhibited autophagy by inhibiting the expression of STAT3 transcription factors in the UUO group.

## 1. Introduction

The prevalence of chronic kidney disease (CKD) is increasing worldwide and fibrosis is responsible for chronic progressive kidney failure [1]. Renal interstitial fibrosis, characterized by the excess deposition of extracellular matrix (ECM) in the tubulointerstitium, is the final common pathway for all progressive forms of CKD, ultimately leading to end-stage renal disease [2]. Other mechanisms, such as oxidative stress, inflammation, mitochondrial damage, endoplasmic reticulum stress, and autophagy, are also involved in the progression of CKD [3]. A previous study found evidence that autophagy in the proximal tubules may promote fibrosis by coordinately activating tubular cell death, interstitial inflammation, and, particularly, the production of pro-fibrotic factors such as fibronectin [2]. Interestingly, depending on the cell or tissue type and the pathological settings, autophagy can be either pro-fibrotic or anti-fibrotic in various organs [4,5,6].

Autophagy is an important process in maintaining homeostasis and is observed in all eukaryotic organisms [7]. Autophagy is the cellular process of the degradation of the cytoplasmic components through the formation of autophagosomes followed by autolysosomes [8]. In this process, phagophore form first, and then expand and fuse to create vesicles called autophagosomes [9]. The autophagosomes eventually fuse with lysosomes, which clear damaged organelles, such as mitochondria, endoplasmic reticulum, and peroxisomes, removing aggregated or misfolded proteins, as well as intracellular pathogens [10]. Microtubule-associated protein light chain 3 (LC3) and Beclin1 are important and dependable markers of autophagy [11]. In fact, LC3 is the most widely used autophagic marker, and it undergoes transformation from the cytosolic form of LC3-I to the lipidated form of LC3-II, indicating autophagosome formation [12]. In addition, Beclin1 is vital in the recruitment of other autophagy-related proteins that play a role in the expansion of pre-autophagosomal membranes and structures [13]. Autophagy can be activated by diverse factors related to the pathogenesis of various diseases, such as neurodegenerative, liver, cardiovascular, and kidney diseases, as well as carcinomas [14,15]. Autophagy in kidney diseases has also been reported [3,16,17].

Of the many signals inducing autophagy, the Janus tyrosine kinase (JAK)/signal transducer and activator of transcription (STAT) is a crucial intracellular signaling pathway that may regulate a variety of inflammatory reactions, proliferations, and differentiations via various cytokines and stimuli [18]. Some studies have shown that this signaling also stimulates excessive cell proliferation, as well as the enhanced production of TGF-β1, collagen IV and fibronectin [19,20]. In addition, the JAK receptor is activated by binding with various cytokines, such as interleukin-6 (IL-6), interleukin-1β (IL-1β), and tumor necrosis factor-α (TNF-α), which phosphorylate the receptor cytoplasmic domain to admit the recruitment and tyrosine phosphorylation of STATs (p-STAT) [21]. Moreover, STAT3 is activated as a potential transcription factor, which mediates the stimulation of renal interstitial fibroblasts and the progression of renal fibrosis in unilateral ureteral obstruction (UUO) mice [22]. Therefore, this study investigated the role of STAT3 decoy oligonucleotides (ODN) in autophagy and their effect in terms of preventing renal fibrosis in kidney disease.

The crosstalk between autophagy and other stress response pathways, including STAT3 signaling, may determine the survival or death of a cell [23]. Qin et al. [24] indicate that STAT3 participates in the process of autophagy and that a variety of cytokines can induce *p*-STAT3 at Ser727, promoting mitochondrial localization and activating autophagy [25]. In addition, STAT3 interacts with autophagy-related proteins in multiple up-regulated autophagy-related genes, such as BECN1, BNIP3, and ATG12 [26], and it stimulates autophagy by up-regulating and stabilizing hypoxia inducible factor-1α (HIF-1α) under hypoxia [27]. Another signaling pathway known to be involved in autophagy regulation is the phosphoinositide 3-kinase (PI3K)/protein kinase B (Akt) pathway [28], which also plays a key role in cell survival, growth, metabolism, and proliferation [29]. In addition, the activation of the PI3K/Akt pathway can phosphorylate the mammalian target of rapamycin (mTOR), which is a major regulator of autophagy and is positively modulated by the PI3K/Akt pathway [28]. Interestingly, the treatment of HeLa cells via starvation or rapamycin, an mTOR inhibitor, has been shown to induce STAT3 activation, which is necessary for the production of IL-6, and promote the proliferation and survival of cancer cells [30]. Persistent autophagy activation is known to enhance kidney function damage [31]. Thus, this study was intended to confirm the association of kidney function and autophagy through the STAT3 decoy ODNs.

Typically, transcription factors are nuclear proteins that play a crucial role in gene regulation and exert either a positive or a negative effect on gene expression. Decoy ODNs, synthetic short DNA segments, are recognized as modulators of transcription factors [32]. They bind to specific consensus sequences of regulatory proteins and transcription factors found in the promoter areas of their target genes [33]. 

This study suppressed the expression of the STAT3 transcription factor synthesized to STAT3 decoy ODNs, which were injected into the tail veins of UUO mice, to explore the regulation of autophagy in UUO-induced renal fibrosis. Therefore, this study investigated the inhibitory effects of STAT3 decoy ODNs on autophagy regulation in an animal model of renal fibrosis.

## 2. Materials and Methods

### 2.1. Synthesis of Decoy ODNs

Decoy ODNs were synthesized by Macrogen Co. Ltd. (Seoul, Korea). Consensus sequences of the STAT3 transcription factor and the scrambled (Scr) decoy ODNs used in this work are listed in Table 1 (the consensus sequences are underlined).

After denaturation lasting 3 min at 95 °C, the ODNs were annealed for 3h as the temperature was gradually decreased to 85 °C. Following the addition of T4 ligase (1U, Takara Inc., Kusatsu, Japan), the ODNs was incubated for 16 h at 16 °C to generate ligated decoy molecules.

### 2.2. Animal Model

Male C57BL/6 mice (6 weeks old, 20–22g; Samtako Inc., Osan, Korea) were housed individually in cages and maintained at set temperature in humidity, and a 12 h light–dark cycle. Then, one week after acclimatization, a total of 35 mice were randomly divided into five groups, as follows: with five mice per group. The first group was the normal control (NC) group; The second group was injected with the STAT3 decoy ODNs (STAT3 group). The third group had UUO surgery (UUO group). The fourth group contained mice that underwent UUO surgery and were injected with the Scr ODNs (Scr group). The fifth group was comprised of mice that underwent UUO surgery and were injected with the STAT3 decoy ODNs (UUO + STAT3 group).

For the UUO surgery, each mouse was anesthetized, its abdominal cavity was incised, and the left ureter was ligated with 5-0 silk suture at both the distal and proximal locations. The STAT3 ODNs (10 μg/μL) and Scr ODNs (10 μg/μL) were injected thrice into the tail veins 2 days before ureteral ligation, and 2 days and 5 days after the UUO operation. A week after the UUO operation, the mice kidneys were collected and studied as described below. The animal protocols were approved by the Institutional Animal Care and Use Committee of the Catholic University of Daegu, Korea (EXP-IRB number: DCIAFCR-190620-07-Y, Approval date: 20 June 2019).

### 2.3. Histologic Analysis

All the collected kidney tissues were fixed in a 10% formalin solution for 24 h at room temperature. The fixed kidney tissues were dehydrated with ethanol, removed with xylene, and placed in paraffin. The paraffin-embedded tissues were cut into 4 μm sections for deparaffinization. The kidney tissue sections were stained with hematoxylin and eosin (H&E), as well as Masson’s trichrome based on standard protocols. All the slides were examined with a Pannoramic^®®^ MIDI slide scanner (3DHISTECH Ltd., Budapest, Hungary).

### 2.4. Immunohistochemical Staining 

Xylene was used for the deparaffinization of the paraffin-embedded tissue sections. They were dehydrated in progressively reducing concentrations of ethanol, and then treated with 3% hydrogen peroxidase in methanol for 10 min to prevent endogenous peroxidase activity. The kidney tissue sections were placed in 10 mM sodium citrate buffer (pH = 6.0) for 5 min at 95 °C. The last step was repeated using a new 10 mM sodium citrate solution (pH = 6.0). The sections were allowed to remain in the same solution while cooling for 20 min, and they were then rinsed in phosphate-buffered saline. The tissue sections were incubated with a primary antibody such as LC3A/B (Cell Signaling Technology, lnc., Beverly, MA, USA; 1:500 dilution), p62 (Abcam, Cambridge, UK; 1:1000 dilution), and Beclin1 (Novus Biologicals, Centennial, CO, USA; 1:400 dilution), for 1 h at 37 °C. The signal was visualized using the EnVision System (DAKO, Carpinteria, CA, USA) for 30 min at 37 °C. As the coloring reagent, 3,30-diaminobenzidine tetrahydrochloride was used, and hematoxylin was used as the counterstain. All the slides were examined by the Pannoramic^®®^ MIDI slide scanner and analyzed with iSolution DT software. The expression levels of the protein were analyzed via the quantification of the captured images, which were examined via slide scanner, with iSolution DT software.

### 2.5. Immunofluorescent Staining

The kidney tissue sections were placed in a blocking serum (5% bovine serum albumin in phosphate-buffered saline) for 1h at room temperature. The tissue sections were incubated with anti-LC3 (Cell Signaling Technology, lnc., Beverly, MA, USA) and fibronectin (Abcam, Cambridge, UK) for 2h at room temperature. Goat anti-mouse and goat anti-rabbit secondary antibodies were conjugated with Alexa Fluor 488 and Alexa Flour 555 (Invitrogen, Thermo Fisher Scientific, Waltham, MA, USA). The tissue sections were stained with the nucleic acid stain Hoechst 33342. The slides were mounted using a mounting medium (DAKO, Carpinteria, CA, USA). The stained slides were viewed under a confocal fluorescence microscope (Nikon, Tokyo, Japan).

### 2.6. Western Blot Analysis

The kidney protein samples were extracted with a lysis buffer (CelLytic™ MT, Sigma–Aldrich, St. Louis, MO, USA). The protein samples were centrifuged at 13,000 rpm at 4 °C for 10 min after incubation on ice for 30 min. Then, the supernatant was collected and the protein concentration was measured using a Bio-Rad Bradford kit (Bio-Rad Laboratories, Hercules, CA, USA) at 595nm using a spectrophotometer. The samples were boiled for 10 min and equal volumes were loaded on precast gradient polyacrylamide gels (Bolt™ 4–12% Bis-Tris Plus Gels; Thermo Fisher Scientific, Waltham, MA, USA) before being transferred to a nitrocellulose membrane (GE Healthcare, Chicago, IL, USA). The membrane was blocked for 2h at room temperature in 5% bovine serum albumin and incubated with primary antibodies (1:1000 dilution) overnight at 4 °C. Horseradish peroxidase-conjugated secondary antibodies (1:1000 dilution) were used in this study. The signal intensity was detected using an image analyzer (ChemiDoc™XRS+, Bio-Rad Laboratories, Hercules, CA, USA) and quantified with Image Lab software (Bio-Rad Laboratories, Hercules, CA, USA). The primary antibodies used in this study were anti-LC3B, anti-SQSTM1/p62, anti-Beclin1, anti- autophagy-related 5–12 (Atg5-12), phospho-STAT3 (Tyr 705), anti-STAT3, anti-GAPDH, anti-phosphor-mTOR (Ser2448), and anti-mTOR (Cell Signaling Technology, lnc., Beverly, MA, USA); anti-Fibronectin, anti-TNF-α, and anti-IL-6 (Abcam, UK) anti-IL-1β, anti-HIF-1α, anti-phospho-PI3K, and anti-PI3K (Santa Cruz Biotechnology lnc., Dallas, TX, USA); and anti-α-SMA (Sigma–Aldrich, St. Louis, MO, USA).

### 2.7. ELISA Assay

The collected mouse serum samples were measured for IL-6 and TNF-α using ELISA Kits (R&D Systems lnc., Minneapolis, MN, USA) according to the manufacturer’s instructions. The kits measured absorbance at 450 nm with a microplate reader. 

### 2.8. Blood Urea Nitrogen, and Creatinine Assays

Blood urea nitrogen (BUN) levels in the mouse serum samples were measured with a BUN Colorimetric Detection Kit (Invitrogen, Thermo Fisher Scientific, Waltham, MA, USA). The assay was performed according to the manufacturer’s protocol, and absorbance was measured at 450 nm. The creatinine levels in the mouse serum samples were measured with a Creatinine Assay Kit (Bioassay systems, Hayward, CA, USA). Absorbance was measured at 510 nm with a microplate reader.

### 2.9. Statistical Analysis

All data were presented as mean ± standard error. Statistical significance was determined via one-way analysis of variance with Tukey’s multiple comparison tests using GraphPad Prism 5.0 (GraphPad Software, Inc., San Diego, CA, USA). Differences with *p* < 0.05 were considered significant.

## 3. Results

### 3.1. Reduction in Renal Interstitial Injury and Fibrosis in UUO Mouse Kidneys Due to STAT3 Decoy ODNs

**Figure 1:**
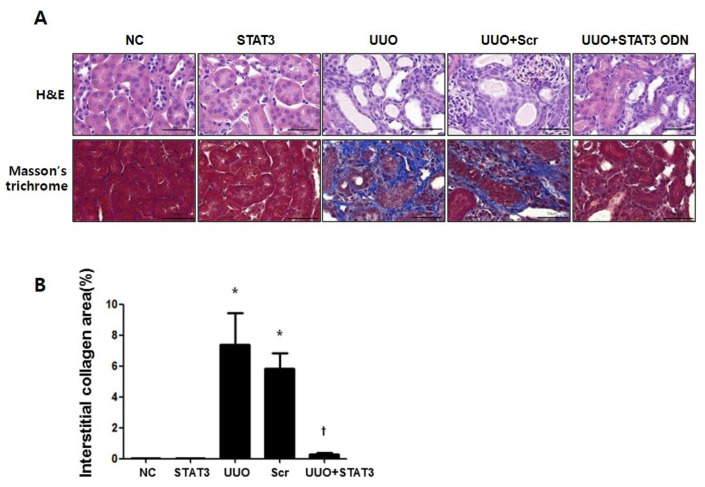
Reduction in Renal Interstitial Injury and Fibrosis in UUO Mouse Kidneys due to STAT3 Decoy ODNs. The effects of STAT3 decoy ODNs on histological alterations after UUO. (**A**) The kidney sections stained with hematoxylin and eosin (H&E) stain and the Masson’s trichrome stain. (**B**) Quantitative analysis of the interstitial collagen area of each group (*n* = 3) and performed at a magnification of 400X. The results are expressed as means ± SE of three independent determinations * *p* < 0.05 vs. NC, † *p* < 0.05 vs. UUO; NC, normal control group; STAT3, STAT3 decoy injection to normal control group; UUO, UUO surgery group; Scr, scramble ODN injection to UUO surgery group; UUO + STAT3, STAT3 decoy ODN injection to UUO surgery group. Abbreviations: UUO, unilateral ureteral obstruction; decoy ODNs, decoy oligonucleotides; STAT3, signal transducer and activator of transcription 3; Scr, scrambled.

This study investigated the effect of STAT3 decoy ODNs on renal interstitial injury and fibrosis in UUO mice kidneys via hematoxylin and eosin (H&E), as well as Masson’s trichrome staining (Figure 1A). The NC group and STAT3 groups showed normal kidney histology. However, glomerular injury was detected and features of severe tubular interstitial injury, including tubular distension and interstitial fibrosis, were observed in the UUO and Scr groups. Compared with the UUO group, the UUO + STAT3 group showed reduced renal damage. The results of Masson’s trichrome staining showed that collagen had accumulated in the UUO group, whereas the expression of collagen was clearly decreased in the UUO + STAT3 group (Figure 1B). Thus, this study showed that glomerulosclerosis and interstitial fibrosis are regulated by STAT3 decoy ODNs in a UUO mouse model.

### 3.2. Inhibition of UUO-Induced Kidney Damage by STAT3 Decoy ODNs

**Figure 2:**
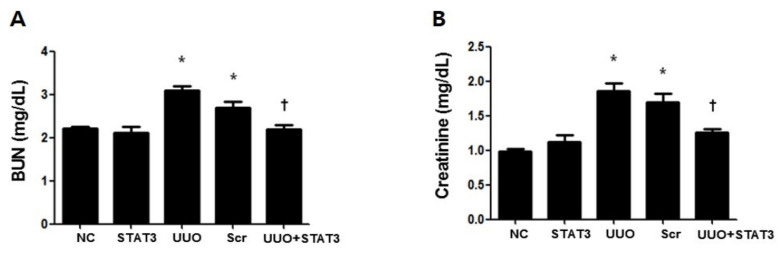
Inhibition of UUO-induced Kidney Damage by STAT3 Decoy ODNs. The effects of STAT3 decoy ODN on level of kidney function after UUO. (**A**) The Urea Nitrogen (BUN) assay, (**B**) the Creatinine analysis. The results are expressed as means ± SE of three independent determinations * *p* < 0.05 vs. NC, † *p* < 0.05 vs. UUO. Abbreviations: UUO, unilateral ureteral obstruction; decoy ODNs, decoy oligonucleotides; STAT3, signal transducer and activator of transcription 3; Scr, scrambled.

To investigate the effect of the STAT3 decoy ODNs in UUO-induced kidney function damage, BUN and creatinine assays were performed using mouse serum samples (Figure 2A,B). Both the UUO and Scr groups showed increased levels as compared with the NC group. However, the STAT3 and NC groups showed similar levels, which were lower compared to that of the UUO group. Therefore, these results demonstrated that the STAT3 decoy ODNs reduced renal damage and prevented renal function damage in UUO mice.

### 3.3. Decrease in Autophagy Expression Due to STAT3 Decoy ODNs in UUO-Induced Renal Fibrosis

**Figure 3:**
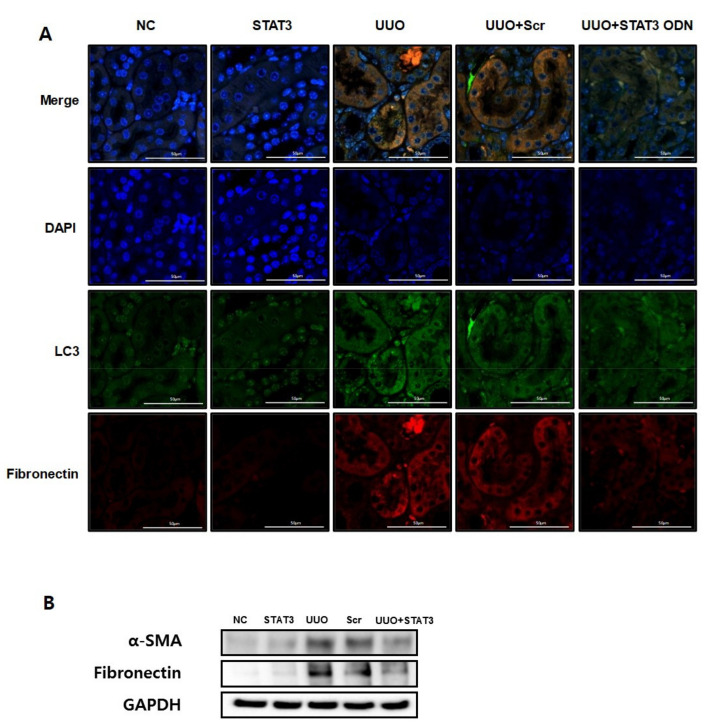
Decrease in Autophagy Expression due to STAT3 Decoy ODNs in UUO-induced Renal Fibrosis. The STAT3 decoy ODN inhibits the expression of fibrotic and autophagy genes in UUO-induced renal fibrosis. (**A**) Immunofluorescence staining shows that LC3 was reduced via STAT3 decoy ODN in UUO-induced renal fibrosis mice. The green color indicates LC3, the red color indicates fibronectin and the blue color indicates the nuclei. (**B**) Western blot analysis shows that STAT3 decoy ODN decreased the expression of fibronectin and α-SMA. Abbreviations: UUO, unilateral ureteral obstruction; decoy ODNs, decoy oligonucleotides; STAT3, signal transducer and activator of transcription 3; α-SMA, α-smooth muscle actin; LC3, microtubule-associated protein light chain 3; DAPI, 4′,6-diamidino-2-phenylindole, Scr, scrambled.

The UUO model is a well-known progressive renal interstitial fibrosis model [34]. This research performed immunofluorescence staining to confirm that autophagy is promoted in UUO-induced renal fibrosis. Fibronectin, a representative ECM protein, is deposited in fibrogenic kidneys [35]. LC3, an autophagy marker, is considered to be a hallmark of autophagy [3]. Therefore, these data confirmed the expression levels of LC3 and fibronectin by staining with different colors (green and red, respectively). As shown in Figure 3A, the expressions of fibronectin and LC3 increased in the UUO group. However, both expressions were decreased in the UUO + STAT3 group. Therefore, this result not only confirms autophagy in UUO-induced renal fibrosis, but also that the STAT3 decoy ODNs suppress autophagy and fibrosis. Additionally, Western blot analysis was performed to confirm the expression of fibrotic genes, such as α-smooth muscle actin (α-SMA) and fibronectin (Figure 3B). The expressions of α-SMA and fibronectin were increased in the obstructive kidneys. In contrast, STAT3 decoy ODNs inhibited α-SMA and fibronectin expressions. Taken together, the inhibition of the STAT3 transcription factor suppresses autophagy and fibrosis in a UUO mouse model.

### 3.4. Regulation of Inflammatory Cytokines in the UUO-induced Renal Fibrosis Model by STAT3 Decoy ODNs

**Figure 4:**
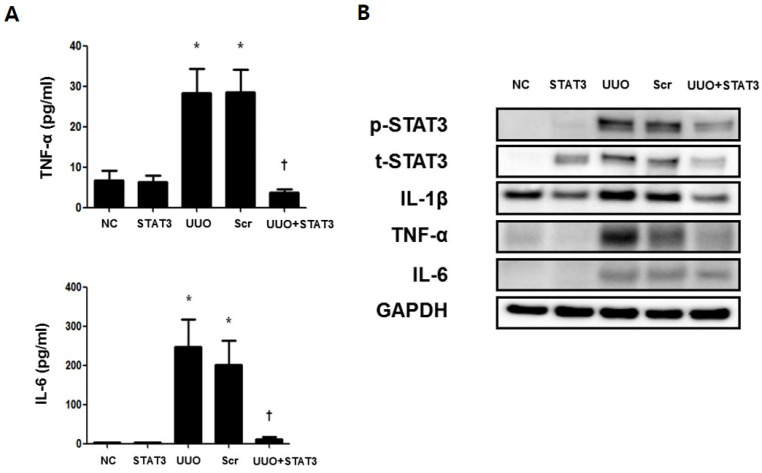
Regulation of Inflammatory Cytokines in the UUO-induced Renal Fibrosis Model by STAT3 Decoy ODNs. The inflammation regulatory effects of STAT3 decoy ODN in obstructive kidney. (**A**). Inhibitory effects of STAT3 on UUO-induced change in serum levels of TNF-α and IL-6 were measured through an ELISA kit (*n* = 5) (**B**) Western blot analysis shows that STAT3 decoy ODN decreased the expressions of inflammatory cytokines such as IL-1β, TNF-α, and IL-6. Western blot results show that STAT3 decoy ODN inhibits the expression of *p*-STAT3. * *p* < 0.05 vs. NC, † *p* < 0.05 vs. UUO. Abbreviations: UUO, unilateral ureteral obstruction; decoy ODNs, decoy oligonucleotides; STAT3, signal transducer and activator of transcription 3; Scr, scrambled; α-SMA, α-smooth muscle actin; IL-1β, interleu-kin-1β; TNF-α, tumor necrosis factor-α; IL-6, interleukin-6.

The activated STAT3 transcription factor is known to induced inflammatory cytokines such as IL-6, TNF-α, and IL-1β [21]. An ELISA assay was performed to confirm the expressions of the inflammatory cytokines IL-6 and TNF-α, in UUO-induced renal fibrosis (Figure 4A). The results revealed increased *p*-STAT3-mediated up-regulated expressions of IL-6 and TNF-α. However, the expression of inflammatory cytokines was inhibited in the UUO + STAT3 group. Moreover, a Western blot test was conducted to confirm the expressions of inflammatory cytokines IL-6, TNF-α, and IL-1β (Figure 4B). The results of the Western blot test showed increased expressions of IL-6, TNF-α, and IL-1β. Furthermore, *p*-STAT3 expression was increased in UUO-induced renal fibrosis. In contrast, the inhibition of the STAT3 transcription factor suppressed the expression of the inflammatory cytokines. Thus, these findings demonstrate that STAT3 decoy ODNs inhibit the expression of STAT3 and inflammatory cytokines.

### 3.5. Regulation Effects of Autophagy by STAT3 Decoy ODNs

**Figure 5:**
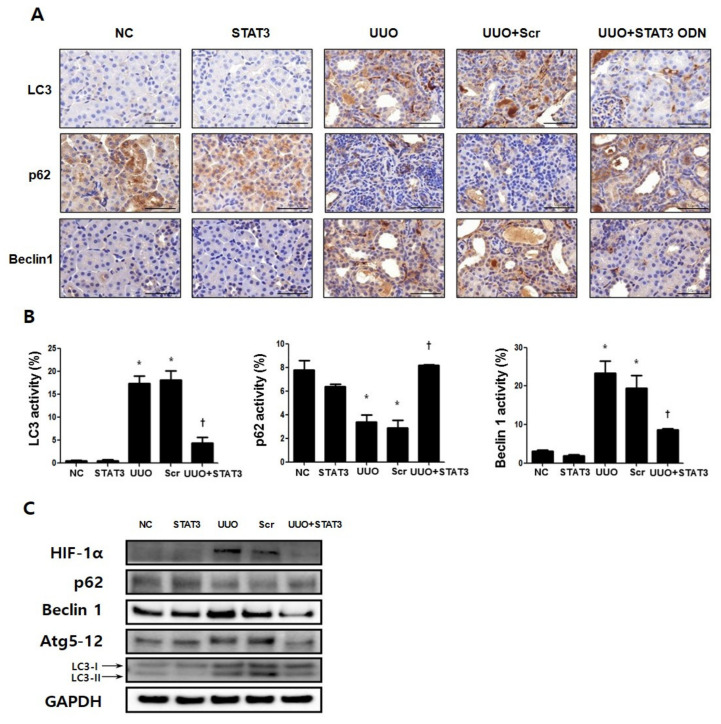
Regulation Effects of Autophagy by STAT3 Decoy ODNs. The STAT3 decoy ODNs suppress autophagy in UUO. (**A**) Immunohistochemical staining shows that autophagy is inhibited by STAT3 decoy ODN. LC3 and Beclin1, autophagy markers, increase in UUO. Autophagosome marker, p62, decreases in UUO. Immunohistochemical staining was used to evaluate the extent of autophagy, which was subsequently quantified (*n* = 3) at a magnification of 400X. (**B**) The graphs show the percentage of LC3, p62 and Beclin1 positive area. (**C**) The representative image of the Western blot analysis shows that STAT3 decoy ODN inhibits autophagy formation. * *p* < 0.05 vs. NC, † *p* < 0.05 vs. UUO. Abbreviations: UUO, unilateral ureteral obstruction; decoy ODNs, decoy oligonucleotides; STAT3, signal transducer and activator of transcription 3; Scr, scrambled; LC3, microtubule-associated protein light chain 3; Atg5-12, autophagy-related 5-12; HIF-1α, hy-poxia inducible factor-1α.

Immunohistochemical staining was performed to investigate the regulatory effects of STAT3 transcription factors in UUO-induced renal fibrosis (Figure 5A). LC3 and Beclin1 are important markers of autophagy [11], and p62 is a known autophagosome marker. Therefore, the expressions of the autophagy-related genes LC3, Beclin1, and p62 in the kidney were confirmed by immunohistochemistry. The results show that the expressions of LC3 and Beclin1 were significantly increased, while that of p62 was decreased in the UUO group. However, the data also showed decreased expressions of LC3 and Beclin1 and increased expression of p62 in the UUO + STAT3 group (Figure 5A,B). In addition, Western blot analysis was performed to confirm the regulation effects of the STAT3 decoy ODNs on autophagy in UUO mice (Figure 5C). Previous research has shown that STAT3 activation induces autophagy by up-regulating HIF-1α and LC3 [36]. In this study, the expressions of HIF-1α, Beclin1, autophagy-related genes 5-12 (Atg 5-12), and LC3 were significantly increased, and the expression of p62 was decreased in UUO-induced renal fibrosis. Additionally, in UUO group, conversion of conversion of the cytoplasmic form of LC3 (LC3-I) to the pre-autophagosomal and autophagosomal membrane-bound form of LC3 (LC3-II) was increased, which means autophagy activity has increased [37]. On the other hand, the HIF-1α, Beclin1, Atg 5-12, and LC3 expressions were reduced, with decreased conversion of LC3-I to LC3-II, and p62 expression was increased in the UUO model injected with STAT3 decoy ODNs. Taken together, these data suggest that the suppression of STAT3 transcription factors can modulate autophagy in UUO-induced renal fibrosis.

### 3.6. Induction of the PI3K-Akt-mTOR Pathway in a UUO Mouse Model with STAT3 Decoy ODN Injection

**Figure 6:**
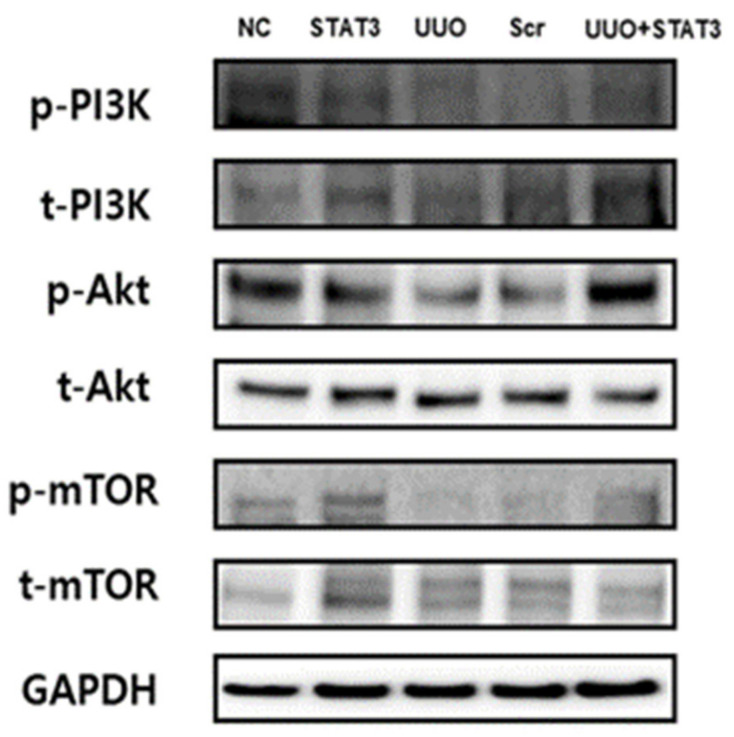
Induction of the PI3K-Akt-mTOR Pathway in a UUO Mouse Model with STAT3 Decoy ODN Injection. The PI3k-Akt-mTOR signaling pathway was increased in UUO mouse model with STAT3 decoy injection. Western blot data show the PI3K-Akt-mTOR pathway. The expression of *p*-PI3K, *p*-Akt, and *p*-mTOR decrease in UUO and autophagy induces in UUO mouse model. Abbreviations: UUO, unilateral ureteral obstruction; decoy ODNs, decoy oligonucleotides; STAT3, signal transducer and activator of transcription 3; Scr, scrambled; PI3K, phosphoinositide 3-kinase; Akt, protein kinase B; mTOR, mammalian target of rapamycin.

The PI3K-Akt-mTOR pathway is well-known in autophagy inhibitory signaling [26]. To identify the molecular mechanism of STAT3 decoy ODNs, PI3K-Akt-mTOR signaling was investigated through Western blot analysis (Figure 6). As in the case of induced autophagy in the UUO group, the expression levels of *p*-PI3K, *p*-Akt, and *p*-mTOR were decreased. However, the same expression levels were found to have increased in the UUO + STAT3 group, similar to the findings for the NC group. Therefore, these data indicate that the STAT3 decoy ODNs inhibit autophagy signaling, and thus, STAT3 transcription factor can be considered as a new therapeutic target for the attenuation of autophagy.

## 4. Discussion

Renal fibrosis is a common pathological result of CKD. Tissue fibrosis is closely associated with chronic inflammation in many pathologies, resulting in functional damage and ultimately leading to terminal renal failure [38]. The progressive property of CKD is associated with the constant loss of renal tissue and its replacement by ECM, culminating in organ fibrosis and failure [39]. The UUO model is a representative animal model of obstructive nephropathy, and it is characterized by progressive tubular interstitial fibrosis [40]. In the UUO model, autophagy is accompanied by increased apoptosis, necrosis, and fibrosis in the renal tubules [41]. Recent studies have shown that dysregulated autophagy is characterized by fibrosis in various tissues, including the cardiac, liver, idiopathic pulmonary, and kidney tissues [42]. However, the role of autophagy in renal fibrosis remains unclear [12]. 

**Figure 7:**
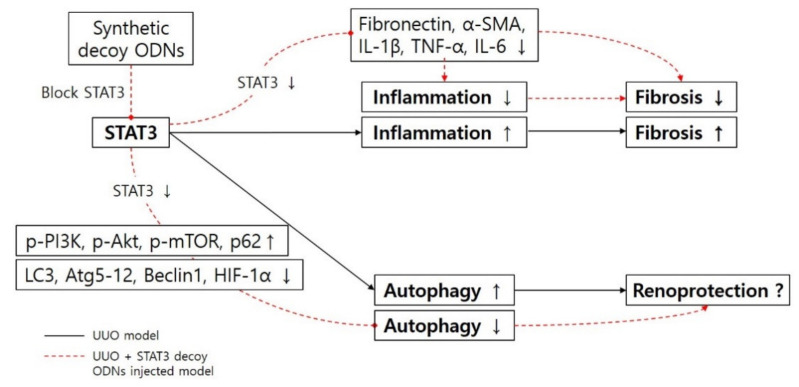
Schematic diagram of the molecular pathway for STAT3 transcription factor and inhibitory effects of synthetic decoy ODNs on autophagy in renal fibrosis. Abbreviations: UUO, unilateral ureteral obstruction; decoy ODNs, decoy oligonucleotides; STAT3, signal transducer and activator of transcription 3; α-SMA, α-smooth muscle actin; IL-1β, interleukin-1β; TNF-α, tumor necrosis factor-α; IL-6, interleukin-6; *p*-PI3K, phospho-phosphoinositide 3-kinase; *p*-Akt, phospho-protein kinase B; *p*-mTOR, phospho-mammalian target of rapamycin; LC3, microtubule-associated protein light chain 3; Atg5-12, autophagy-related 5-12; HIF-1α, hypoxia inducible factor-1α.

The autophagy induced by UUO play a renoprotective role in the kidney [17,43]. Generally, the level of autophagy is increased in damaged tissue. It also has the ability to protect kidneys. In our study, fibrosis was inhibited when STAT3 was suppressed with synthetic decoy ODNs. It has been reported that STAT3 is transcriptional factor which is increased in the process of inflammation [44]. In addition, according to Bromberg et al. [45], STAT3 is associated with fibroblast transformation. It is believed that intravenously injected synthetic decoy ODNs suppressed inflammation of UUO kidneys, which resulted in the suppression of renal fibrosis in this study, though this was not our intention (Figure 7). This study focused on STAT3 transcription factors, which are known to play a role in the regulation of autophagy and fibrosis. The results of this research confirm autophagy regulation via STAT3 decoy ODNs in a UUO-induced renal fibrosis model.

Some studies have shown that autophagy induces fibrosis in the liver. Thoen et al. [46] demonstrated that autophagy induced in hepatic stellate cells can decompose lipids and thus stimulate the activation of these cells to promote liver fibrosis. In contrast, autophagy in hepatic macrophages and hepatocytes suppresses inflammation and apoptosis, thus preventing liver fibrosis [5]. However, autophagy and fibrosis signaling in the kidney remains unclear. Therefore, this study investigated the signaling pathway of autophagy, and suggested that the STAT3 transcription factor induces both fibrosis and autophagy.

The JAK/STAT signaling pathway is a vital intracellular signaling pathway involving various cytokines such as IL-6, IL-1β, and TNF-α, which may regulate a variety of inflammatory reactions, proliferations, and differentiations [18]. Moreover, receptor binding is activated in the JAK/STAT pathway, and STAT3 activation mediates the stimulation of renal interstitial fibroblasts and the progression of renal fibrosis in UUO mice [47].

Past research has also provided increasing evidence for the role of STAT3 in the regulation of autophagy [48,49]. However, some studies have indicated that STAT3 mediates an inhibitory effect on autophagy [24,37], while others have presented conflicting results [50,51]. Thus, STAT3 is associated with regulating autophagy, although its precise role is unclear [52]. Therefore, this study investigated whether STAT3 mediates autophagy.

Some studies have reported that persistent autophagy activation may induce renal cell death pathways and lead to kidney damage [31,53]. As one of transcription factors in autophagy regulation, STAT3 reportedly increases *p*-STAT3 expression to mediate autophagy [26]. To confirm this finding, this study investigated whether the STAT3 transcription factor mediates autophagy and kidney damage. Therefore, STAT3 decoy ODNs were synthesized to inhibit the STAT3 transcription factor and thus suppress of autophagy. Additionally, ODNs with consensus binding sequences for specific transcription factors have been studied regarding the regulation of gene expression in living cells [32]. Decoy ODNs are transcription repressors, that also bind to the transcription factor, inhibiting gene expression by occupying the DNA binding site of the transcription factor in the nucleus [54]. Thus, this study confirmed the regulation of autophagy expression with STAT3 decoy ODNs in a UUO mouse model. The results showed that autophagy was suppressed by the STAT3 decoy ODNs and damage to kidney function was prevented. 

As mentioned above, STAT3 is a stress response pathway that has been implicated in several steps of the autophagy process, but its role is further complicated by its cellular localization [27]. For example, nuclear STAT3 has ability to contribute to autophagy inhibition. Cytoplasmic STAT3 inhibits autophagy via a direct effect on EIF2AK2 activity. On the other hand, cytoplasmic STAT3 activation can mediate the pro-autophagic response through autophagy-related proteins, such as EIF2A, FOXO1, and FOXO3 [27]. Additionally, STAT3 is a signaling molecule and transcription factor that participates in other pathways and the STAT3 blockade effect may be linked to some of its other genomic and non-genomic actions. This is why there are some studies with results that are contrary to the results of our study. Further studies are mandatory.

This work also investigated the down-regulation of autophagy via STAT3 decoy ODNs. Immunohistochemistry and Western blot analyses were performed to confirm autophagy expression in UUO-induced renal fibrosis via the expression levels of LC3, Beclin1, and p62. Kang et al. [55] observed that IL-6 and the overexpression of STAT3 promote autophagic flux in pancreatic tumor cells by enhancing LC3 turnover, suggesting that the IL-6/*p*-STAT3 pathway is a positive regulator of autophagy in pancreatic cancer. Similar to Kang et al., this study regulated the STAT3 transcription factor so as to inhibit autophagy in mouse kidneys, and the results showed that the expressions of LC3 and Beclin1 increased while that of p62 decreased, in a UUO-induced kidney fibrosis model. Inhibition of the STAT3 transcription factor group down-regulated LC3, whereas the expression of p62 was up-regulated. This result indicates that the STAT3 transcription factor regulates autophagy. Some previous works have shown that STAT3 activation enhances autophagy by up-regulating the expression levels of HIF-1α and LC3 [36]. Therefore, the Western blot test was conducted in this research project to determine whether the STAT3 decoy ODNs suppress HIF-1α expression and autophagy. The results suggested that HIF-1α is down-regulated by the STAT3 decoy ODNs, leading to autophagy inhibition.

Autophagy can be regulated by various signaling pathways, including the PI3K/Akt pathway. In addition, the activation of the PI3K/Akt pathway can phosphorylate mTOR, which is a major regulator of autophagy [28]. In fact, mTOR inhibits autophagy under growth-promoting conditions [10]. Li et al. [11] demonstrated that autophagy increased the expression levels of *p*-PI3K, *p*-Akt, and *p*-mTOR, and suppressed those of LC3-II and Beclin1. Similar results were obtained in the present study via Western blot analysis, confirming that the expression levels of *p*-Akt, *p*-PI3K, and *p*-mTOR decreased in the UUO-induced renal fibrosis group. In contrast, the expression levels of *p*-Akt, *p*-PI3K, and *p*-mTOR increased in the UUO + STAT3 group. These findings suggest that the STAT3 decoy ODNs inhibit autophagy expression by activating the PI3K/Akt/mTOR pathway.

In summary, the synthetic non-coding RNA targeting STAT3 transcription factor, which is known to induce fibrosis and inflammatory response, was newly synthesized for this study. This work confirmed that renal fibrosis and autophagy were induced in a UUO mouse model. The down-regulation of fibrosis and autophagy was induced after the injection of the STAT3 decoy ODNs. The STAT3 decoy ODNs also suppressed inflammation. When the STAT3 transcription factor was inhibited by the STAT3 decoy ODNs, kidney damage decreased in the UUO + STAT3 group. However, the prevention of kidney damage was attributed to fibrosis inhibition, inflammation inhibition, and autophagy inhibition. Therefore, this study showed that the inhibition of the STAT3 transcription factor with synthetic decoy ODNs in a UUO mouse model suppressed autophagy and prevented kidney damage. Additionally, this research confirmed that STAT3 decoy ODNs decreased HIF-1α expression and increased PI3K-Akt-mTOR signaling activity in a UUO mouse model. Thus, autophagy was suppressed by the inhibition of the STAT3 transcription factor in a UUO mouse model. Taken together, these results suggest that STAT3 decoy ODNs may be involved in the regulation of autophagy and fibrosis, and that, thus, they are a promising new therapeutic target in CKD.

## Figures and Tables

**Table 1:** Target sequences of the decoy ODNs used in this study.

Decoy	Sequence
*Scr*	5′-*GAATTCAATTCAGGGTACGGCAAAAAATTGCCGTACCCTGAATT*-3′
*STAT3*	5′-*GAATTCCCTTCCCGGAATTAAAAAATTCCCGGAAGG*-3′

## Data Availability

Not applicable.
